# Everybody's Page

**Published:** 1891-01-10

**Authors:** 


### Everybody's Page.

To all letters soliciting his subscription to anything Erskine had a regular form of reply, which ran as follows: "Sir, I feel much honoured by your application to me, and I beg to subscribe"—here the reader had to turn over the leaf—"myself your very obedient servant," &c.

The action of the Leicester Town Council in deciding, though only by a majority of one, after two nights' discussion, to open the Free Library, Art Gallery, and Museum on Sunday for twelve months as an experiment, will be approved by the Progressists as much as it will be deplored by the Puritanical party, but its success or failure will be watched with equal interest by all parties, though with different feelings.

The supply of petroleum in different parts of the world is so great and practically inexhaustible that the time cannot be very remote when it will, in many cases, take the place of coal. If only this liquid fuel, which burns without smoke or smell, were used in some of our great factories and on the underground railway, we might have a very much pleasanter atmosphere to breathe in our large cities, where the air is now always full of sulphur and carbon.

No doubt London ratepayers would hail with delight a regulation to the effect that no children should be sent to school unless showing a decided inclination for study as a happy issue out of their afflictions, for it would mean the closing of all schools. Does it follow that a boy who has an aversion to study will keep steadily at work in some useful calling, and have not the most idle boys at school frequently turned out excellent citizens? A bookworm's life is seldom useful—a prig's is highly objectionable.

Our forefathers had queer diseases and queer remedies. For a "chin-cough"—whatever that may have been—the following was considered to be a sure cure: "Take a spoonful of wood-lice, bruise them, mix them with breast milk, and take for three or four mornings as you find benefit. It will cure, but some must take it longer than others." If it is to be our evil destiny to be inflicted with "chin-cough," and modern science can find no other cure, let the fell disease do its worst; we prefer death to cure.

It appears that many old, and now disused, gold mines give evidence of a knowledge of mining on the part of the ancients little, if at all, inferior to that of the present day. In some respects our forefathers seem to have been distinctly in advance of us, for there are traces of shafts having been started and galleries driven, where modern miners, notwithstanding all the appliances science has invented for them, hesitate to begin work. Perseverance was clearly not one of the qualities lacking with those who have gone before us, however deficient they may have been in scientific knowledge.

Doctor Fordyce sometimes drank a good deal at dinner. He was summoned one evening to see a lady patient, when he was more than half seas over, and conscious that he was so. Feeling her pulse, and finding himself unable to count its beats, he muttered, "Drunk, by jove!" Next morning, recollecting the circumstance, he was greatly vexed, and just as he was thinking what explanation of his behaviour he should offer to the lady, a letter from her was put into his hand. "She too well knew," said the letter, "that he had discovered the unfortunate condition in which she was when he last visited her; and she entreated him to keep the matter secret, in consideration of the enclosed (a hundred pound bank-note)."

The British public will be singularly wanting in gratitude if they have failed to appreciate the munificent generosity at Christmas time of some of the southern railway companies. There is something truly magnificent in the liberality of the arrangement making the return half of tickets for distances under ten miles taken on Christmas Day available on the following day.

The fact that the injurious and noxious particles which form the component parts of a London fog can be, and apparently are, wafted forty miles into the country is a most unpleasant one for our friends who have hitherto congratulated themselves that it is not their lot to live in the metropolis. But it is a pardonable selfishness on the part of long-suffering Londoners to wish that the fogs could be wafted off more rapidly.

The adoption by the vestries in London of the practice of spreading salt on the snow in the streets and on the pavements has called forth some strong protests. The practice is not only objectionable on the ground that it turns the roads into an almost impassable quagmire of slush, but the mixture of salt and snow has a most unpleas[illegible] penetrating quality which ruins the boots of everyone and the temper of most who suffer from the pain of chilblains, for which they get so little sympathy.

The following recipe for plum pudding, which appeared recently in the *Times*, will be found a useful one to the numerous people who can ill afford to pay twopence each for eggs and one shilling a pound for sultana raisins: "Take a quarter of a pound each of suet, flour, and brown sugar, one pound of dates, and a quarter of a grated nutmeg. Chop the suet finely, stone and cut up the dates, and mix all the ingredients well together, moistening with as little water as possible; boil the whole in a buttered basin for four hours." This pudding will be found rich, nourishing, and wholesome, and resembling closely the ordinary plum pudding; enough for six persons can be made for the moderate cost of fourpence, with dates at twopence a pound.

The extraordinary case of hydrophobia in a horse which occurred recently at Dover, and was the subject of inquiries by the Board of Agriculture, shows very clearly the necessity of stringent regulations with a view to stamping out this terrible disease The dog which was the cause of the horse's agonizing death also attacked several sheep, which were at once destroyed, and is reported to have scratched several people, and yet, with a clear knowledge of the dire mischief that can be caused by one dog, there are people sufficiently foolish and so hopelessly sentimental as to prate idiotically about the inhumanity of muzzling dogs. And how jubilantly these people will bleat now that the muzzling order has been withdrawn in London!

If the London public take exception to some of the doings of their School Board, they cannot fail to be grateful to the Board for its endeavour to co-operate with the medical officers of health with a view to the adoption of some systematic and uniform method within the metropolitan area by which they can prevent more effectively the spread of infectious diseases. It is undoubtedly of the greatest importance that the medical officer to the School Board should be made aware of the extent of infectious disease within the metropolis, and that he should be furnished weekly with a copy of the return of any infectious diseases within the sanitary areas of the town. The diseases which are to be dealt with under any scheme which may ultimately, and we hope speedily, be adopted are scarlet fever, small-pox, diphtheria, membraneous croup, enteric, typhoid, and typhus.

